# Reddit Discussions During the 2022 Mpox Outbreak: Observational Analysis of Sentiment, Topics, and Audience Engagement

**DOI:** 10.2196/90152

**Published:** 2026-06-23

**Authors:** Xi Ning Luo, Zahra Movahedi Nia, Jude Dzevela Kong

**Affiliations:** 1 Artificial Intelligence and Mathematical Modeling Lab, Dalla Lana School of Public Health, University of Toronto, 155 College St, Room 500, Toronto, ON, M5T 3M7, Canada, 1 416 978 0901; 2 Department of Computer Science, Faculty of Arts and Science, University of Toronto, Toronto, ON, Canada; 3 Global South Artificial Intelligence for Pandemic and Epidemic Preparedness and Response Network, Toronto, ON, Canada; 4 Department of Mathematics and Statistics, Faculty of Science, York University, North York, ON, Canada; 5 Africa-Canada Artificial Intelligence and Data Innovation Consortium, Toronto, ON, Canada; 6 Munk School of Global Affairs and Public Policy, University of Toronto, Toronto, ON, Canada; 7 Institute of Health Policy, Management and Evaluation, University of Toronto, Toronto, ON, Canada; 8 Department of Mathematics, Bahen Centre for Information Technology, University of Toronto, Toronto, ON, Canada

**Keywords:** audience engagement, monkeypox, virus, infectious outbreak, Reddit, social media, sentiment analysis, topic modelling, natural language processing, public discourse

## Abstract

**Background:**

Public health crises often reshape online discourse by amplifying uncertainty, frustration, stigma, and misinformation, with important implications for risk communication.

**Objective:**

This study examines these dynamics on Reddit (Reddit Inc) during a recent outbreak, using Mpox as a case study.

**Methods:**

We analyzed sentiment, topical themes, and audience engagement in posts and comments drawn from 4 Mpox-related subreddits. Using natural language processing methods, we applied sentiment analysis and latent Dirichlet allocation to classify 1169 posts and 6571 comments (from July 21, 2021, to July 16, 2025) into sentiment categories and 9 distinct topics. Of the 1169 posts, 611 (52.3%) were neutral, 370 (31.6%) were negative, and 188 (16.1%) were positive. Among comments, 2825 of 6571 (43%) were neutral, 1962 (29.9%) were negative, and 1784 (27.1%) were positive. We then used Kruskal-Wallis tests, Dunn post hoc comparisons, and Vargha-Delaney A to assess relationships among sentiment, topic, and engagement metrics.

**Results:**

Engagement differed significantly by sentiment (*P*<.001) and topic (*P*<.001). Negative posts had higher median scores (median 7, IQR 2-27) than positive ones (median 5, IQR 2-16; *z* score=6.02; adjusted *P*<.001; Vargha-Delaney A=0.55). Posts about systemic public health failures (Topic 4) received lower median scores (median 4, IQR 1.75-14.25) than other topics. Topic 9 accounted for 980 of 6571 (14.9%) comments, dominating discussions regardless of original post topic. Positive posts generated 284 of 922 (30.8%) positive comments, whereas negative posts received 526 of 1615 (32.6%) negative comments. Comments on positive posts had higher sentiment scores (Vargha-Delaney A=0.550), whereas comments on negative posts had lower sentiment scores (Vargha-Delaney A=0.463). Topic-level differences in comment sentiment were also observed: comments responding to posts on scientific- and policy-related debates (Topic 8) were more positive (Vargha-Delaney A=0.531), whereas those on systemic failures (Topic 4) were more negative (Vargha-Delaney A=0.478).

**Conclusions:**

Overall, the findings highlight how audience reactions can amplify emotionally charged narratives and reframe technical information into socially and politically charged debates. These insights can inform public health communication strategies by anticipating likely audience responses, mitigating stigma and misinformation, and fostering constructive dialogue during health crises.

## Introduction

Public health crises often reshape online discourse by amplifying uncertainty, frustration, stigma, and misinformation, with important implications for risk communication. Social media platforms—and Reddit (Reddit Inc) in particular—have become crucial spaces where the public seeks information, shares experiences, and reacts to evolving guidance during outbreaks. In this context, Mpox serves as a timely case study for understanding how online discussions unfold during a public health crisis.

Mpox, formerly known as Monkeypox, is a double-stranded DNA virus in the Orthopoxvirus genus, which contains smallpox, cowpox, and vaccinia. The disease first became endemic in the Democratic Republic of Congo in 1970 and for decades remained a rare zoonotic disease confined to Central and West Africa, mainly affecting children in rural areas. Over time, cases steadily increased, with a notable outbreak in the United States in 2003 linked to imported rodents. By the 2010s, outbreaks were reported in multiple African countries, with a major resurgence in Nigeria in 2017-2018 that also led to exported cases abroad. Since May 2022, however, Mpox has evolved into a global outbreak affecting over 70 countries, driven primarily by human-to-human transmission and presenting with atypical clinical features compared to earlier outbreaks [1].

The rapid global spread of Mpox has not only posed clinical and epidemiological challenges but has also fueled widespread uncertainty, frustration, stigma, and misinformation online. As seen in other public health crises, public discourse around outbreaks is often shaped by polarized sentiments, conspiracy theories, and shifting attitudes toward prevention measures. Social media platforms, in particular, have become crucial spaces for both the dissemination of health information and the circulation of misleading narratives. Past studies have shown how online discussions can capture nuanced public responses during pandemics. For instance, an analysis of Canadian city-based subreddits revealed that local sentiment toward COVID-19 vaccines varied across regions, with joy being the most associated emotion in vaccine-related conversations [2]. Within the context of Mpox, large-scale analyses of X (formerly Twitter; X Corp) and Facebook (Meta Platforms, Inc) posts found that discussions frequently linked the outbreak to lesbian, gay, bisexual, transgender, queer, and more (LGBTQ+) communities, with 8 of 10 identified topics actively stigmatizing sexual minorities [3]. Similarly, thematic analysis of Mpox-related Reddit posts in LGBTQ+ subreddits highlighted changes in sexual behavior, concerns about vaccine safety, and the negative mental health effects of stigma, while also showing how these communities relied on online platforms for information and mutual support [4]. Expanding on these findings, another study analyzed Reddit comments between June 1, 2022, and August 5, 2022, and identified dominant themes such as symptoms, transmission, vaccination, government interventions, and homophobia [5].

Beyond Reddit, deep learning and network-based analyses have shown that misinformation and online toxicity were defining features of Mpox-related discourse. One study mapped Twitter narratives and found that misleading or irrelevant information spread faster and reached wider audiences than official communications [6]. Likewise, another study identified widespread toxic discourse on Twitter centered on homophobia, racism, and politicization, illustrating how stigma and polarization were amplified during the outbreak [7]. Complementary qualitative research also emphasized the dual role of social media as both an effective communication tool and a challenge for public health response. One paper reported that social media platforms in the United Kingdom facilitated rapid information sharing and advocacy during the Mpox outbreak but simultaneously reproduced exclusionary dynamics and misinformation [8]. Similarly, another found that public health departments’ Twitter communications promoted vaccination and community engagement but struggled to maintain visibility amid competing misinformation streams [9]. Collectively, these studies underscore both the potential and pitfalls of social media in shaping public understanding and communication during health crises.

While prior studies on Mpox have illuminated the thematic structure and emotional tone of Mpox discussions online, they rarely consider how different forms of audience engagement, such as upvotes, comments, shares, or views, interact with the sentiment and topics of the original content. This is a critical omission, as engagement metrics are not monolithic but reflect distinct audience behaviors. A study on Facebook news posts [10] demonstrated that commenting, sharing, and liking are separate modes of engagement, each driven by different content characteristics and emotional triggers. For example, politics-related posts were most likely to generate comments, while health-related posts were strongly associated with sharing, and positive-tone posts received more likes. Emotional triggers also mattered, with anger increasing commenting and sharing, while sadness encouraged sharing and reacting. However, less is known about how these dynamics manifest on Reddit during public health crises—particularly how engagement relates to the sentiment and topical framing of outbreak-related posts and how audiences respond through comments. Using Mpox as a case study, this work examines how the relationship between posts and audience reactions may shape discourse during a health crisis.

To address this gap, this study investigates two research questions (RQs):

1. How do audience engagement metrics relate to the sentiment and topic categories of social media posts about health emergency crises (Mpox as a case study)?
2. How do audience responses (comment, sentiment, and topical focus) differ for posts of varying sentiments and topic categories (Mpox as a case study)?

By bridging this gap, this study provides a more nuanced understanding of how online discussions evolve from the initial post to audience interaction, offering insights that can help public health communicators design more effective messaging, guide platform moderators in mitigating stigma and misinformation, and support vulnerable communities in accessing accurate information during public health crises.

## Methods

### Data Collection and Cleaning

The data used for this study were gathered from Reddit. Four subreddits with sufficient size and general discussions related to Mpox were selected: r/Monkeypox, r/monkeypoxpositive, r/worldnews, and r/news [11-14]. The 4 subreddits were selected to capture both specialized and general-audience discourse. r/Monkeypox and r/monkeypoxpositive focus specifically on outbreak-related discussions, whereas r/news and r/worldnews provide broader news-driven conversations and public reactions. This combination allowed examination of both community-specific and general public engagement. Other subreddits (eg, r/PublicHealth and r/Coronavirus) were not included because Mpox-related content appeared sporadically and was less consistently represented during preliminary screening.

This dataset was collected from July 2021 to July 2025 to capture discourse preceding the global outbreak, the peak transmission period in 2022‐2023, and subsequent post-peak discussions, allowing examination of how sentiment and thematic emphasis evolved across emergency and later phases.

Since r/worldnews and r/news also contain discussions about other topics, relevant posts were filtered using the keywords “mpox” and “monkeypox.” These keywords reflect the most commonly used labels during the study period. While related terms (eg, orthopoxvirus or strain-specific names) may have captured additional posts, preliminary searches indicated that such terminology appeared infrequently in general news subreddits.

By using ReddAPI (SeasonedCode; available through RapidAPI, Nokia) [15], posts and their respective comments were retrieved from these 4 subreddits. Key information retrieved for posts included subreddit, permalink, title, description, score (calculated by the number of upvotes minus downvotes), and created time. Key information retrieved for comments included subreddit, permalink, permalink of the parent post, text message, score, and created time.

Data processing and analysis were done using R (version 4.5.1; R Foundation for Statistical Computing) within RStudio Desktop (version 2025.05.1+513; Posit Software) [16,17]. Posts with empty entries in any column were removed, with the exception of description, which is optional on Reddit. Comments containing any empty entries were also excluded. After removing missing values, 1491 posts and 7706 comments remained. Language detection was then performed using the detect_language() function from the Compact Language Detector 3 library, which predicts the most likely language of each text string and returns “Not Applicable” if the language cannot be reliably determined. Language detection was applied to post titles and full comment bodies. English was identified as the dominant language, accounting for 1186 posts and 6802 comments. Only English-language content (language code “en”) was retained for analysis.

### Sentiment Analysis Methods

A sentiment score was assigned to each post and comment using the sentimentr library [18], which performs sentence-level scoring and accounts for contextual valence shifters such as negators, amplifiers, deamplifiers, and adversative conjunctions (eg, “but”). This approach was selected over simpler bag-of-words lexicons because Reddit posts and comments often contain mixed sentiment within a single message, informal language, and variable length. A general-purpose lexicon-based method was therefore considered appropriate for capturing contextual sentiment shifts in heterogeneous social media text. The sentimentr package computes sentence-level polarity scores using a lexicon-based approach that incorporates valence shifters (eg, negators such as “not,” amplifiers such as “very,” and adversative conjunctions) to adjust contextual sentiment intensity. Sentence-level scores are aggregated to produce a document-level sentiment score for each post or comment. Higher values indicate more positive sentiment, values near zero reflect neutrality, and lower values indicate more negative sentiment. Based on these scores, each observation was also assigned a categorical sentiment label: scores below −0.1 were classified as “negative,” scores above 0.1 as “positive,” and scores in between as “neutral.” The thresholds of 0.1 and −0.1 were selected to distinguish substantively polarized text from near-neutral language, as very small absolute scores typically represent minimal lexical polarity.

### Topic Modeling Methods

**Figure 1.**
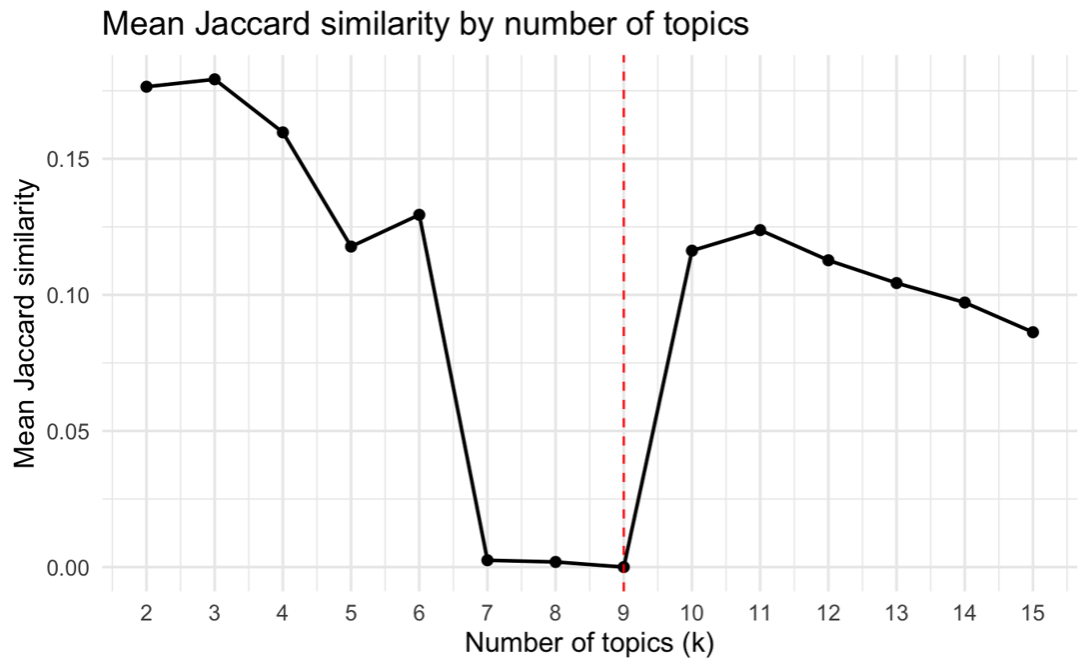
Mean Jaccard similarity among the top 10 terms of each topic as a function of the number of topics (k). For each candidate model (k=2-15), latent Dirichlet allocation (LDA) was fitted, and the average pairwise Jaccard similarity between the top terms of topics was computed. Lower values indicate greater separation between topics.

Topic modeling was conducted using the topicmodels library [19,20]. The topicmodels package implements probabilistic topic modeling algorithms, including latent Dirichlet allocation (LDA), which models each document as a mixture of topics and each topic as a probability distribution over words. Before modeling, posts (title and description) and comments were combined into a single corpus and preprocessed using standard text normalization procedures, including lowercasing, punctuation removal, number removal, stop-word removal, and whitespace trimming. A document-term matrix was then constructed, and sparse terms were removed to reduce noise. Documents with no remaining terms after preprocessing were excluded. LDA was applied to identify clusters of topics from the text data. For each document, LDA estimates a topic probability distribution (gamma matrix), and each document was assigned to the topic with the highest posterior probability. To determine the optimal number of topics, the k-value (the number of topics) was fine-tuned by testing values between 2 and 15. For each k-value, topic-word distributions (beta matrix) were extracted, and pairwise Jensen-Shannon divergence (JSD) was computed between topics. The mean JSD among the top 10 terms was then calculated, with higher values indicating more distinct topics, and the k-value with the highest mean JSD (k=9) was selected, as shown in Figure 1.

After statistical topic assignments were finalized, a large language model (LLM; Gemini 1.5 Flash; Google) was used solely to generate human-readable topic summaries. The LLM did not influence clustering or document assignment. For each topic, randomly sampled subsets of posts and comments were provided to the model across multiple runs. Across iterations, approximately 575‐669 unique messages per topic were examined, representing the majority of documents assigned to each topic. Generated summaries were reviewed and aggregated to produce final topic descriptions. This process focused on interpretive labeling rather than altering statistical topic assignments. The exact prompt structure used for topic labeling is described below.

The first step was a system-level instruction. The following system message was provided before any topic text was sent:

You are helping analyze Reddit discussions of Mpox. I have grouped Reddit posts/comments into statistically distinct topics. I will now send you several chunks of text per topic. Each chunk will be clearly labeled. Do not respond yet — just read and remember the content. At the end, I will ask for a summary of each topic’s main theme.

The second step was topic-specific text input. For each topic, 50 randomly sampled documents (posts and comments combined) were selected. The full text of each sampled document was provided. Each topic was labeled and structured as follows:

=== Topic X Chunk 1 ===

[Full text of sampled posts and comments belonging to Topic X]

No summarization or preprocessing beyond earlier text cleaning was performed before submission to the model.

The third step was a first-stage summarization instruction. After all topic-specific text had been provided, the following instruction was issued:

I finished sending all the Reddit text. Now summarize the main theme of each topic (1 to 9) in one clear, concise sentence.

Format exactly as: Topic 1: [theme]; Topic 2: [theme]; … Topic 9: [theme]

Focus on the most distinctive ideas across the chunks for each topic.

The fourth step was a multi-run stability procedure. To reduce variability in single-model outputs, the above procedure was repeated 20 times using newly sampled documents for each topic. This resulted in 20 candidate summaries per topic.

The final step was the second-stage consolidation prompt. All 20 auto-generated summaries for each topic were then provided back to the model using the following instruction:

I am studying discussions related to Mpox. Here are 20 auto-generated theme summaries for Topic X:

[Theme 1]

[Theme 2]

…

[Theme 20]

Now summarize the main theme of each topic (1-9) based on the summaries I sent.

Format your response as:

Topic 1 theme: [theme]; Topic 1 concise summary: [2‐3 words];

Topic 2 theme: [theme]; Topic 2 concise summary: [2‐3 words];

…

Each ‘theme’ should be a complete sentence capturing the topic’s overall meaning. Each ‘concise summary’ should be 2‐3 keywords.

The final theme labels were generated from this consolidation stage and subsequently reviewed by the research team to ensure conceptual clarity and distinguishability across topics. No manual reassignment of documents was performed. A small number of observations could not be assigned to a topic due to formatting issues, and these were removed from the analysis.

### Statistical Analysis Methods

After processing, a total of 1169 posts and 6571 comments were used for the analysis. Both the earliest and latest creation dates for posts and comments are July 21, 2021, and July 16, 2025, respectively. Exploratory data analysis was then carried out using a combination of visualizations and summary tables to identify initial patterns and trends in the data. Subsequent analyses focused on derived attributes of each post or comment, such as sentiment scores, sentiment labels, assigned topic categories representing the main subject of the text, and engagement metrics, rather than on the full textual content itself. To assess differences in score distributions across sentiment labels and topics, the Kruskal-Wallis test was applied. The Dunn test and Kolmogorov-Smirnov statistics were used for pairwise comparisons to more comprehensively evaluate the extent of differences between groups. Nonparametric statistical tests were selected due to the nonnormal distribution and skewness observed in engagement metrics and sentiment scores. The Kruskal-Wallis test was used to compare differences across multiple independent topic groups, as it does not assume normality. When overall group differences were significant, the Dunn test with appropriate adjustment was applied for pairwise comparisons. The Kolmogorov-Smirnov test was used to assess distributional differences between groups, as it is sensitive to differences in both central tendency and distribution shape. All statistical tests were considered significant at *P*<.05, roughly equivalent to 2 standard deviations from the mean [21].

### Post Content Divergence Analysis Methods

Pairwise comparisons were further evaluated using the Vargha-Delaney A (stochastic superiority, “A12”) statistic, which estimates the probability that a randomly selected score from one group will exceed that from another. This measure was applied to compare scores across sentiment and topic groups, as well as to examine sentiment differences in comments associated with posts of varying sentiment and topic. This approach was chosen due to the skewed, nonnormal distribution of engagement and sentiment scores. The Vargha-Delaney A statistic was calculated as a nonparametric effect size measure, quantifying the probability that values from one group exceed those from another. The threshold for statistical significance was set at 0.05 for all tests [21].

### Ethical Considerations

This study adhered to widely accepted ethical standards governing online research and used only publicly accessible data obtained from Reddit. Safeguarding user privacy was a central consideration throughout the data collection process. Information was sourced exclusively from public Reddit communities (subreddits), where posts and discussions are openly visible and are not shared under any expectation of confidentiality. To minimize intrusion, the collection procedures were automated and structured to remove identifying details from the dataset. No personal identifiers, including usernames, profile details, or IP addresses, were retained at any stage. Consequently, the final dataset consisted solely of anonymized comment text, posting dates and times, and the associated subreddit, ensuring that no individual users could be identified.

## Results

### Sentiment Analysis Results

**Figure 2.**
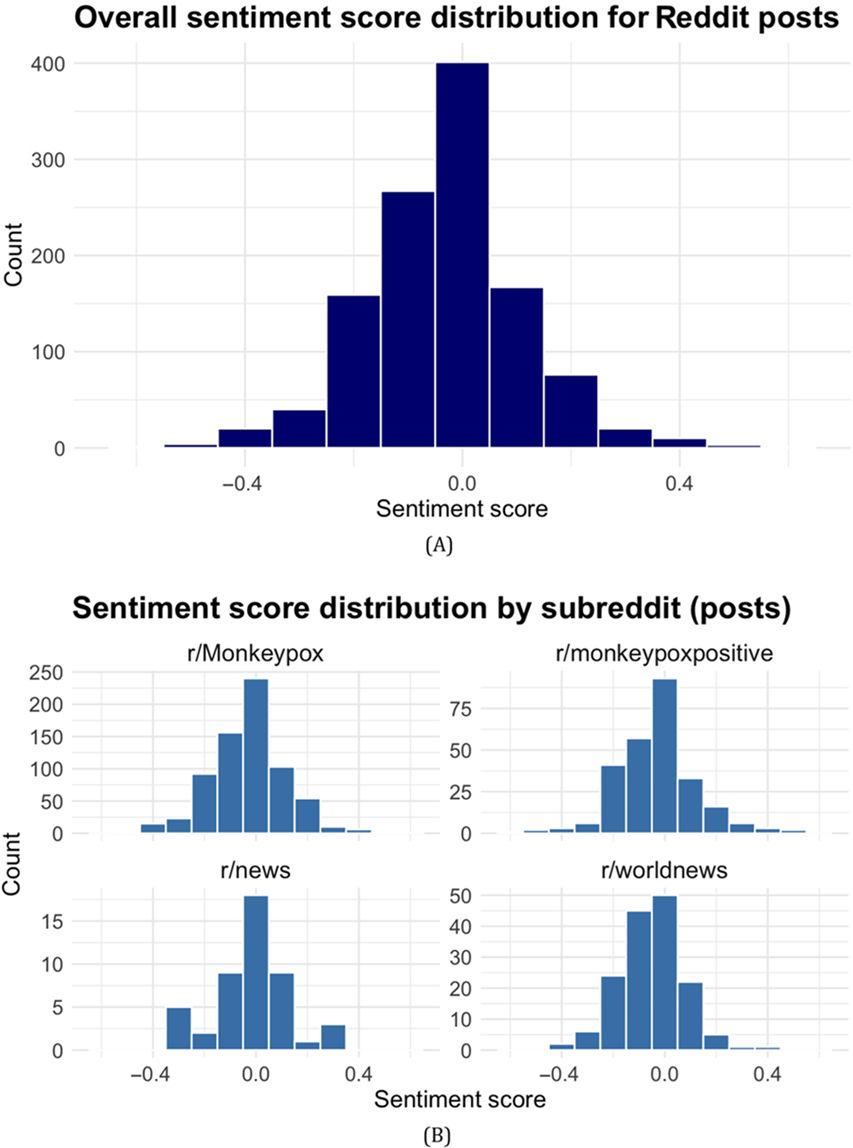
Distribution of sentiment scores (A) for posts in the entire dataset and (B) for each of the 4 subreddits.

The distribution of sentiment scores for Reddit posts is shown in Table 1 and Figures 2A and 2B. Across all posts, the mean sentiment score (−0.0326, SD 0.1450) was nearly neutral and slightly on the negative side, with a median close to zero (−0.0120, IQR −0.120 to 0.0404). The overall distribution was approximately symmetric and centered around zero, with a slight right skew as shown in Figure 2. When disaggregated by subreddit, r/Monkeypox [11] and r/monkeypoxpositive [12] showed similar distributions, while r/worldnews [13] posts tended to have more negative sentiment on average (mean −0.051, SD 0.128).

**Table 1.** Summary statistics of sentiment scores for posts in the entire dataset and for each of the 4 subreddits.

Subreddit	Count	Mean (SD) sentiment	Median (IQR) sentiment	Range (min-max) sentiment
ALL	1169	−0.0326 (0.1452)	−0.0120 (−0.1260 to 0.0431)	−0.5924 to 0.6114
r/Monkeypox [11]	703	−0.0309 (0.1458)	0.0000 (−0.1294 to 0.0483)	−0.5349 to 0.6114
r/monkeypoxpositive [12]	263	−0.0290 (0.1518)	−0.0199 (−0.1205 to 0.0404)	−0.5924 to 0.5093
r/news [14]	47	−0.0189 (0.1517)	0.0000 (−0.1106 to 0.0631)	−0.3408 to 0.3455
r/worldnews [13]	156	−0.0505 (0.1277)	−0.0461 (−0.1321 to 0)	−0.4184 to 0.3719

**Figure 3.**
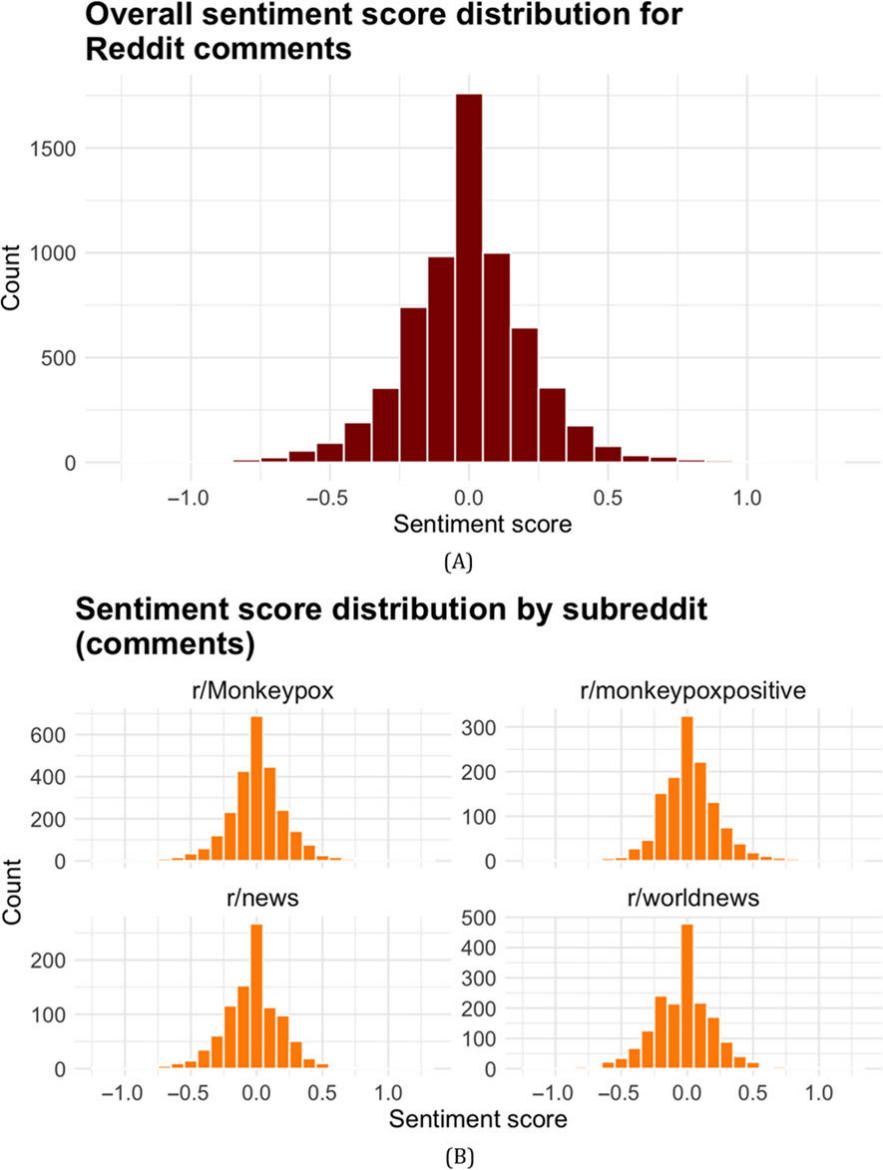
Distribution of sentiment scores (A) for comments in the entire dataset and (B) for each of the 4 subreddits.

For comments, summary statistics are provided in Table 2, with distributions shown in Figures 3A and 3B. Overall, comments also centered around zero (mean −0.006, SD 0.2263; median 0, IQR −0.1335 to 0.1158), but their distribution appeared more symmetric than that of posts as shown in Figure 3A for the whole dataset and Figure 3B for each of the 4 subreddits. The SDs of scores were higher, reflecting greater variation in comment sentiment. Subreddit-level distributions followed a similar pattern, with comments from r/Monkeypox [11] and r/monkeypoxpositive [12] exhibiting slightly more positive tendencies compared to r/news [14] and r/worldnews [13].

**Table 2.** Summary statistics of sentiment scores for comments in the entire dataset and for each of the 4 subreddits.

Subreddit	Count	Mean (SD) sentiment	Median (IQR) sentiment	Range (min-max) sentiment
ALL	6571	−0.0060 (0.2263)	0.0000 (−0.1335 to 0.1158)	−1.2478 to 1.3112
r/Monkeypox [11]	2572	0.0048 (0.2196)	0.0000 (−0.1114 to 0.1182)	−1.2478 to 1.3112
r/monkeypoxpositive [12]	1270	0.0238 (0.2175)	0.0000 (−1.090 to 0.1364)	−0.9550 to 1.0606
r/news [14]	964	−0.0287 (0.2240)	0.0000 (−0.1520 to 0.0970)	−1.2300 to 0.8573
r/worldnews [13]	1765	−0.0307 (0.2393)	0.0000 (−0.1746 to 0.1021)	−1.1976 to 1.0062

For Reddit posts, 611 of 1169 (52.3%) posts were neutral, followed by 370 (31.6%) negative and 188 (16.1%) positive posts. This suggests that although most posts adopted a neutral tone, negative sentiment was expressed about twice as often as positive sentiment. For Reddit comments, the distribution followed a similar pattern but with a greater proportion of positive content compared to posts. Neutral comments were most common, with a count of 2825 out of 6571 (43%), followed by 1962 (29.9%) negative and 1784 (27.1%) positive comments. Compared to posts, comments contained relatively less neutral and more positive sentiment.

### Topic Modeling Results

A total of 9 distinct topics were identified through topic modeling:

1. Discussions focus on global Mpox transmission concerns, public health responses (especially vaccine distribution and messaging), and stigma, with emphasis on LGBTQ+ communities and personal experiences of symptoms or issues. Sample text:

Post: Monkeypox, COVID, and Racism... Journalists and editors of global North media outlets badly need training on how to not be racist and stigmatizing in their reporting [of] Ebola, COVID-19, monkeypox, tweeted Dr. Madhu Pai…

Comment: I’m a week past my last TPOXX dose and my anorectal lesions are still healing. They are getting better every day but it’s just taking a while. Externally it looks pretty much back to normal but I can still feel the internal ones. Definitely will not be bottoming for at least another week or two. No pain when having a bowel movement, at least.

2. Debates center on Mpox spread among MSM, vaccine access challenges, and comparisons to COVID-19, alongside personal symptom accounts and frustration with public health failures. Sample text:

Post: The CDC Scientist Who Couldn’t Get Monkeypox Treatment

Comment: Wasn’t the vaccine two dose vaccine? How effective will it be now that you’re only getting 20% of the original vaccine? How long before it becomes effective against the virus? The original dosage you get protection after 14 days of the second dose.

3. Postpeak concerns include declining cases, lingering public health challenges (eg, misinformation and isolation fatigue), and scientific analyses of mutations and long-term impacts. Sample text:

Post: WHO recommends gay and bisexual men limit sexual partners to reduce the spread of monkeypox

Comment: I’m so sorry you’re feeling this and hopefully it ends soon (and doctors do something.) I’m following your story and thank you so much for sharing this, even though it’s awful. Sadly people need the awareness that we can ALL get this.

4. Criticism of systemic public health failures (eg, vaccine disparities and bureaucratic delays) and anxiety about broadening transmission (eg, pediatric cases and workplaces). Sample text:

Post: Thailand says mpox case recorded in foreigner traveling from Africa

Comment: Now I’m expecting a rightwing conspiracy claim that humans cannot catch monkey pox, it is a hoax to trick people into believing in evolution.

5. Fears of Mpox resurgence or endemicity, debates over vaccine strategies, and critiques of global disparities in outbreak response. Sample text:

Post: Two people diagnosed with monkeypox in London, health officials say

Comment: Sure. Remind me how many vaxs are the US giving? 50,000?

6. Emergence of high-risk Mpox variants (eg, Clade I), underreported global spread, and debates over containment strategies beyond MSM communities. Sample text:

Post: Monkeypox Can Spread In Three Ways. Here’s What To Know And How To Avoid Infection.

Comment: But we are not shutting down the sex clubs for 2 weeks to flatten the curve?

7. Cross-border spread of strains, personal accounts of severe symptoms, and critiques of institutional response delays and inequities. Sample text:

Post: Is this mpox? Day of 3 since I noticed this pimple-like bump on my thigh. Not feeling any of the known mpox symptoms but the bump looks suspicious :(

Comment: What good is 40,000 doses in a country with 80+ million?

8. Scientific- and policy-related debates on mutations and reinfection, stigma’s role in public perception, and advocacy for marginalized groups amid systemic gaps. Sample text:

Post: Sex workers are hit hard by Congo’s mpox outbreak but say their only option is to keep working

Comment: somehow smallpox, a more contagious and deadlier cousin, was contained. sounds like motivated reasoning from so-called experts

9. Sociopolitical reactions dominate, including stigma, naming controversies, and comparisons to past pandemics, alongside frustration with institutional mismanagement. Sample text:

Post: As Chicago’s Market Days approaches, doctors and organizers share monkeypox concerns, advice

Comment: This little gem caught me by surprise with the level of accuracy and simplicity that their guide manages to achieve in describing mpox to clients who may be affected by it or interested in it as a result of living in group homes or congregate settings, and working or living independently with close caretakers. Compassionate, clear and concise. Kudos to ASAN. I’m impressed that someone put this much effort into developing materials like this specifically about mpox for a vulnerable, population.

The distribution of posts and comments across these topics is provided in Table 3. Across all subreddits, posts were most frequently associated with Topic 8, which accounted for 371 posts. This was followed by Topic 7 with 203 posts. By contrast, Topic 4 was the least represented among posts, with only 39 entries. For comments, the distribution was somewhat different. Topic 9 dominated with 980 comments, followed by Topic 3 with 865 comments and Topic 4 with 809 comments. In comparison, Topic 8, which was the most common for posts, contained only 465 comments, the lowest among all topics.

Subreddit-level distributions revealed further variation. On r/Monkeypox [11] and r/monkeypoxpositive [12], posts again concentrated in Topic 8, while comments were more evenly distributed, with Topics 3, 4, and 9 consistently drawing higher engagement. In contrast, r/news [14] and r/worldnews [13], which contained fewer total posts, showed a relatively weaker emphasis on Topic 8.

**Table 3.** Number of posts and comments per topic for each subreddit and the whole dataset.

Topic no	r/Monkeypox		r/monkeypoxpositive		r/news		r/worldnews		Whole dataset	
	Posts	Comments	Posts	Comments	Posts	Comments	Posts	Comments	Posts	Comments
1	75	306	21	119	7	112	24	190	127	727
2	45	277	22	125	2	122	9	192	78	716
3	40	325	21	233	3	118	16	189	80	865
4	23	308	4	259	5	54	7	188	39	809
5	35	296	16	127	8	102	16	214	75	739
6	90	235	12	117	4	109	13	198	119	659
7	134	263	39	76	8	109	22	163	203	611
8	225	241	110	63	5	61	31	100	371	465
9	36	321	18	151	5	177	18	331	77	980

### Relationships Between Engagement, Sentiment, and Topic

**Figure 4.**
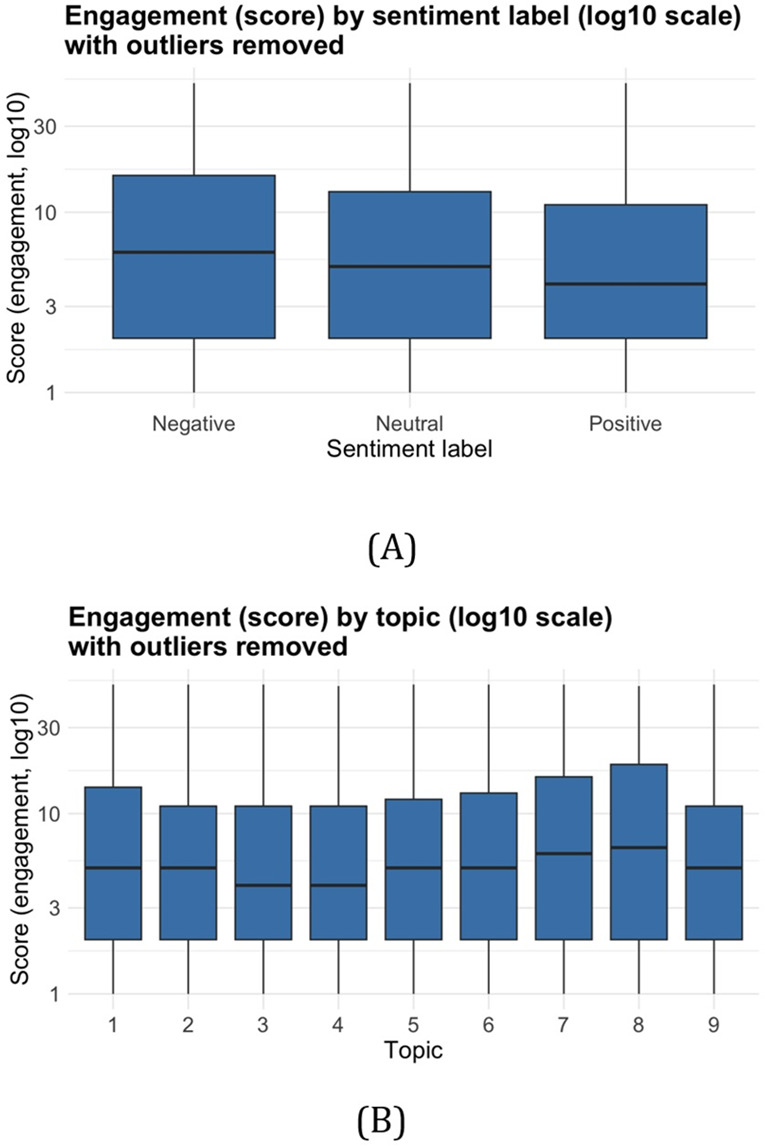
Distribution of engagement scores by (A) sentiment label and (B) topic. Engagement scores are displayed on a log10 scale to account for the highly right-skewed distribution. Boxplots show median and IQRs, with points representing individual posts and comments. A total of 1052 outliers were excluded based on the 1.5×IQR criterion before plotting.

Engagement scores were highly right-skewed, with a small number of posts receiving disproportionately high levels of engagement. To improve interpretability, Figures 4A and 4B display engagement on a log10 scale. Despite this skewed distribution, clear differences in median engagement were observed across both sentiment labels and topics.

The Kruskal-Wallis test confirmed that engagement scores differed significantly across sentiment and topic categories for the dataset overall, as shown in Table 4. At the subreddit level, patterns varied: on r/Monkeypox [11], both sentiment and topic distributions differed significantly, while on r/monkeypoxpositive [12], no significant differences were observed. On r/news [14], engagement varied only by sentiment label, whereas on r/worldnews [13] differences were significant only by topic.

**Table 4.** Kruskal-Wallis test results of score distributions for sentiment label and topic.

Subreddit	Variable	*P* value	Count
ALL	Sentiment Label	<.001^a^	7740
ALL	Topic	<.001^a^	7740
r/Monkeypox [11]	Sentiment Label	<.001^a^	3275
r/Monkeypox [11]	Topic	<.001^a^	3275
r/monkeypoxpositive [12]	Sentiment Label	.52	1533
r/monkeypoxpositive [12]	Topic	.54	1533
r/news [14]	Sentiment Label	.02^b^	1011
r/news [14]	Topic	.36	1011
r/worldnews [13]	Sentiment Label	.61	1921
r/worldnews [13]	Topic	<.001^a^	1921

a *P*<.001.

b *P*<.05.

**Figure 5.**
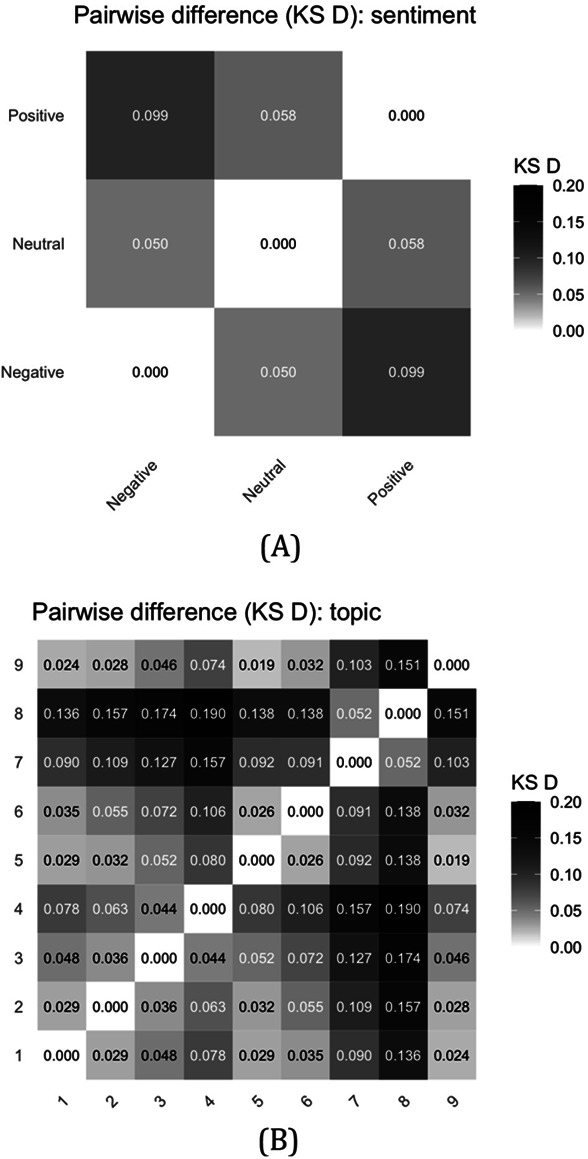
Pairwise Kolmogorov-Smirnov statistics comparing score distributions across (A) sentiment labels, and (B) topics. KS D: Kolmogorov-Smirnov D.

Pairwise comparisons provided additional insights. Tables5  6 show Dunn test results for pairwise score distribution differences among sentiment labels and topics, respectively. These results include *z* scores and Benjamini-Hochberg adjusted *P* values. Only statistically significant (*P*<.05) comparisons are reported in Table 6. Dunn test revealed that posts with positive and negative sentiment differed the most in score distributions, while neutral content showed moderate differences compared to both groups, according to Table 5. Topic 8 differed in distribution the most compared to other topics, according to Table 6. Kolmogorov-Smirnov statistics indicated that Topics 7 and 8 stood out, exhibiting the largest differences in score distributions from other topics, although they were relatively similar to each other, as provided in Figures 5A and 5B for the sentiments and the topics, respectively.

**Table 5.** Pairwise comparison of sentiment scores (Dunn test).

Group 1	Count 1, n	Median (IQR) 1	Group 2	Count 2, n	Median (IQR) 2	*z* score	*P* value adjusted
Negative	2332	7.0 (2-27)	Positive	1972	5.0 (2-16)	6.0217	<.001
Neutral	3436	5.5 (2-22)	Positive	1972	5.0 (2-16)	3.7881	<.001
Negative	2332	7.0 (2-27)	Neutral	3436	5.5 (2-22)	2.8774	.004

**Table 6.** Pairwise comparison of topic scores (Dunn test).

Group 1	N 1	Median (IQR) 1	Group 2	N 2	Median (IQR) 2	*z* score	*P* value adjusted
3	945	4 (2-16)	8	836	10 (2-37.25)	−6.7162	<.001
4	848	4 (1.75–14.25)	8	836	10 (2-37.25)	−7.9924	<.001
1	854	5 (2-21)	8	836	10 (2-37.25)	−5.6745	<.001
2	794	5 (2-19)	8	836	10 (2-37.25)	−5.8514	<.001
5	814	5 (2-20)	8	836	10 (2-37.25)	−5.0353	<.001
8	836	10 (2-35.25)	9	1057	5 (2-20)	5.7662	<.001
3	945	4 (2-16)	7	814	8 (2-31)	−4.9035	<.001
4	949	4 (1.75–14.25)	7	814	8 (2-31)	−6.2184	<.001
6	778	6 (2-19)	8	836	10 (2-37.25)	−4.7381	<.001
2	794	5 (2-19)	7	814	8 (2-31)	−4.1211	<.001
1	854	5 (2-21)	7	814	8 (2-31)	−3.9130	<.001
7	814	8 (2-31)	9	1057	5 (2-20)	3.9131	<.001
5	814	5 (2-20)	7	814	8 (2-31)	−3.2993	.002
4	848	4 (1.75-14.25)	6	778	6 (2-19)	−3.0922	.005
6	778	6 (2-19)	7	814	8 (2-31)	−3.0240	.006
4	848	4 (1.75–14.25)	5	814	5 (2-20)	−2.8855	.008
4	848	4 (1.75–14.25)	9	1057	5 (2-20)	−2.6605	.02
1	854	5 (2-21)	4	848	4 (1.75-14.25)	2.3403	.04

**Figure 6.**
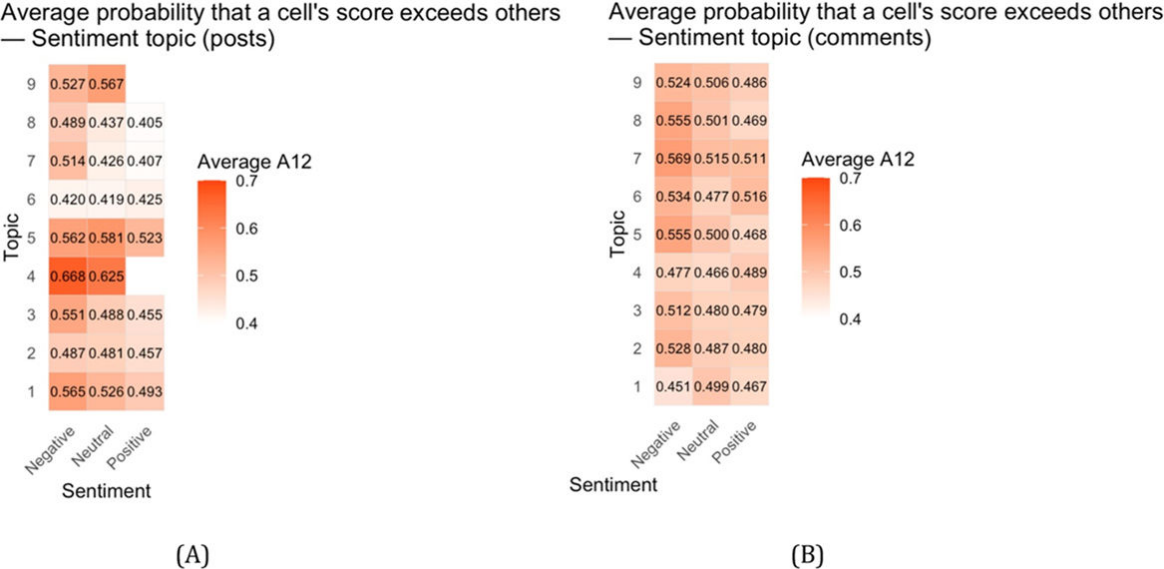
Vargha-Delaney A, comparing scores across different sentiment and topic combinations for (A) posts and (B) comments.

Vargha-Delaney A statistics, which estimate the probability that a random score from one group exceeds that of another, highlighted systematic trends, as shown in Figures 6A and 6B for the posts and the comments, respectively. Across all topics, negative posts tended to attract higher engagement than positive ones, with this trend particularly prominent for posts. Among posts, Topic 4 achieved the highest scores on average, while Topic 6 had the lowest. In contrast, for comments, Topics 7, 5, and 8 attracted the highest scores, whereas Topic 1 and Topic 4 had the lowest.

### Divergence Between Post Content and Audience Responses

**Figure 7.**
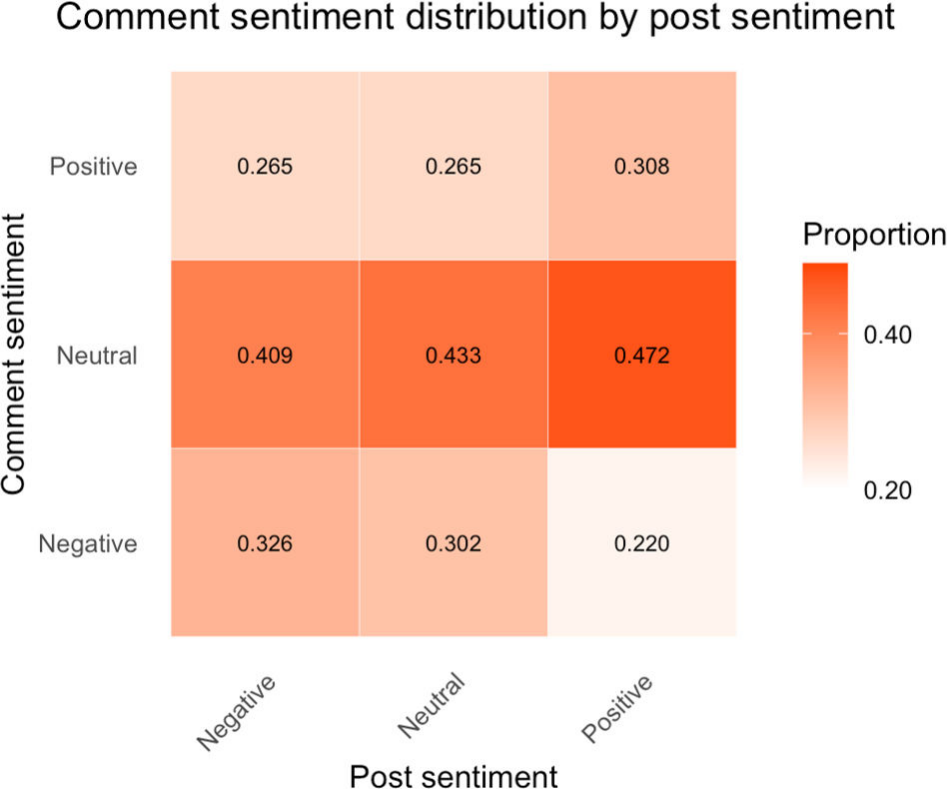
Heatmap showing the proportion of comments in each sentiment label category for posts of different sentiment categories.

The relationship between post sentiment and the sentiment of associated comments is shown in Figure 7. Across all post types, neutral comments made up the majority of responses, though patterns varied with post sentiment. Positive posts tended to elicit a greater proportion of positive comments (284/922 comments, 30.8%), while negative posts attracted relatively more negative comments (526/1615 comments, 32.6%). Neutral posts drew a more balanced distribution of comment sentiments, with neutral comments remaining dominant (1194/2756 comments, 43.3%). Vargha-Delaney A statistics confirmed these patterns, showing that comments on positive posts had significantly higher sentiment scores, while comments on negative posts tended to be lower, as shown in Table 7.

**Table 7.** Vargha-Delaney A comparing comment sentiment scores for posts of different sentiment labels.

Post sentiment category	A vs others, mean (SD)	A vs others, median (IQR)	Cliffs delta vs others, mean (SD)	Dominance share
Positive	0.5502 (0.0111)	0.5502 (0.5463-0.5542)	0.1005 (0.0222)	1.0
Neutral	0.4870 (0.0416)	0.4870 (0.4723-0.5017)	−0.0260 (0.0222)	0.5
Negative	0.4627 (0.0295)	0.4627 (0.4523-0.4732)	−0.0745 (0.0589)	0.0

Comment sentiment also varied by the topic of the original post, as shown in Table 8. Posts classified under Topic 8 elicited the highest overall comment sentiment scores, followed by Topics 2 and 1. In contrast, comments from posts on Topics 4 and 6 had the lowest sentiment scores, suggesting that discussions centered on systemic failures or new risks provoked more negative responses.

**Table 8.** Vargha-Delaney A comparing comment sentiment scores for posts of different topics.

Post sentiment label	A vs others, mean (SD)	A vs others, median (IQR)	Cliffs delta vs others, mean (SD)	Dominance share
8	0.5305 (0.0113)	0.5312 (0.5226-0.5380)	0.0609 (0.0225)	1.000
2	0.5140 (0.0152)	0.5167 (0.5085-0.5241)	0.0281 (0.0304)	0.875
1	0.5112 (0.0154)	0.5140 (0.5056-0.5208)	0.0223 (0.0308)	0.750
5	0.5019 (0.0151)	0.5059 (0.4911-0.5123)	0.0038 (0.0303)	0.625
9	0.4999 (0.0153)	0.5032 (0.4895-0.5107)	−0.0002 (0.0307)	0.500
7	0.4906 (0.0155)	0.4909 (0.4812-0.5028)	−0.0187 (0.0310)	0.375
3	0.4883 (0.0152)	0.4894 (0.4788-0.4988)	−0.0234 (0.0303)	0.250
6	0.4857 (0.0147)	0.4860 (0.4771-0.4965)	−0.0285 (0.0293)	0.125
4	0.4778 (0.0138)	0.4804 (0.4689-0.4887)	−0.0444 (0.0276)	0.000

**Figure 8.**
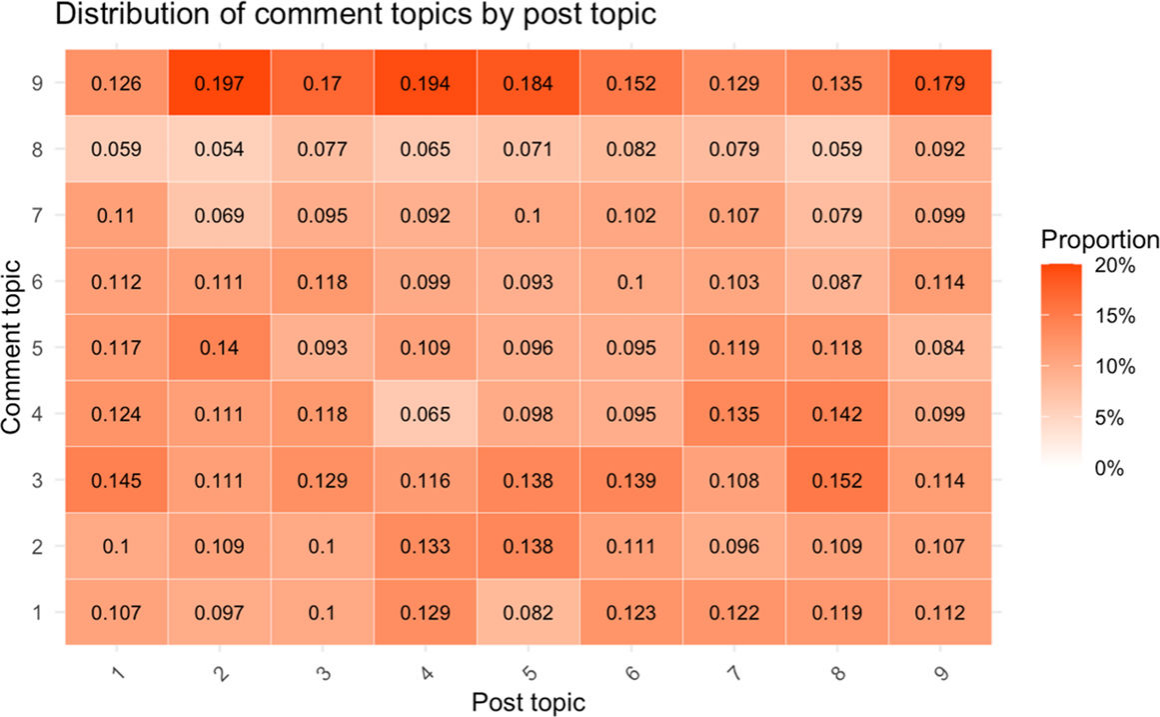
Heatmap showing the proportion of comments of each topic for posts of different topics.

Thematic alignment between posts and comments was further examined in Figure 8. Comment topics did not always mirror post topics, indicating that discussion often shifted focus. Regardless of the post topic, Topic 9 was the most common theme among comments. This reflects a broader trend of audiences reframing technical or health-focused posts into socially and politically charged conversations.

## Discussion

### Principal Findings

This study analyzed the topics and sentiments of Reddit posts regarding Mpox. Topic 9, which centers on sociopolitical reactions including stigma, naming controversies, and comparisons to past pandemics, dominated comments across the dataset. This heightened engagement may reflect multiple factors. First, the platform’s culture likely encourages discussion and debate, particularly around topics with social or ethical implications. Second, sociopolitical and stigma-related content in health crises often elicits emotional responses, personal anecdotes, and opinion sharing, making these posts more likely to attract comments. Finally, attributes specific to the Mpox outbreak, such as attention to vulnerable populations, institutional mismanagement, and ongoing public discourse about naming and stigma, may have contributed to the higher comment volume. These factors together help explain why Topic 9 generated more discussion than other topics. This research examined how audience engagement metrics relate to the sentiment and topic types of Reddit posts about Mpox (RQ 1) and how the focus of the audience, reflected in comment sentiment and topic, varies in response to posts of different sentiment and topic types (RQ 2). By linking original posts with the comments they generated, this study advances understanding of how public discourse around health crises evolves on social media platforms.

### Relationships Between Engagement, Sentiment, and Topic

The analysis revealed that engagement scores were highly skewed, with a small number of posts receiving disproportionately high upvotes. Despite this skew, clear differences emerged across sentiment and topic categories. Negative posts tended to attract higher engagement than positive ones, and posts about systemic public health failures (Topic 4) and sociopolitical reactions (Topic 9) garnered especially high scores. By contrast, posts emphasizing technical or epidemiological details (Topic 6) received comparatively lower engagement.

These findings echo the past observation that engagement is not a uniform behavior but is shaped by specific content and emotional triggers [10]. Just as a previous study [10] found that emotionally charged political posts tended to elicit more active engagement than neutral ones, the present results suggest that negative sentiment and controversial or blame-oriented topics captured more attention on Reddit. This underscores the role of emotional salience in driving user reactions.

### Divergence Between Post Content and Audience Responses

Although neutral comments were most common across all types of posts, positive posts elicited relatively more positive comments, and similarly, negative posts drew more negative comments. Furthermore, comments on posts about systemic failures (Topic 4) and high-risk variants (Topic 6) were more negative, whereas comments on posts about scientific and policy debates (Topic 8) tended to be more positive.

Thematic alignment between posts and comments was loose: comment topics often shifted away from the original post’s topic, and sociopolitical reactions (Topic 9) dominated the comment space regardless of post topic. This pattern resonates with previous research on Mpox discourse, which has documented the tendency for online discussions to become infused with stigma and broader sociopolitical framings, especially around LGBTQ+ communities [3,4]. While prior studies primarily focused on the content of posts, these results extend that understanding by showing how audience responses can reframe even technically focused or neutral posts into socially charged debates.

These findings also suggest that emotionally charged and blame-oriented narratives may play a role in shaping audience engagement and subsequent discussion trajectories. Posts emphasizing institutional failures, perceived inequities, or stigmatized groups often attracted more polarized responses, which in turn redirected conversations toward broader sociopolitical concerns. Even when original posts focused on epidemiological updates or technical information, comment threads frequently introduced themes related to accountability, stigma, or policy critique. This dynamic indicates that audience engagement may not only amplify emotionally salient content but also actively reshape the framing of discussions beyond the initial informational focus.

### Comparison With Previous Works

Our findings align with recent studies examining Mpox-related discourse on social media, which have reported predominantly neutral-to-negative sentiment and the presence of stigma-related narratives [3,22-26]. Prior analyses of Twitter and Reddit data during the 2022 outbreak observed that discussions frequently focused on transmission risk [22,27], vaccine access [5], and the association of Mpox with LGBTQ+ communities [3,28,29], often accompanied by misinformation and stigmatizing language [3,30-32]. These studies similarly noted that emotionally charged content tended to receive greater engagement, reinforcing the visibility of polarizing narratives [4].

The topic modeling results in this study extend previous work by identifying distinct thematic clusters spanning epidemiological updates, policy debates, and sociopolitical reactions. Earlier research has emphasized that public health discussions during outbreaks often evolve from technical information sharing to broader debates about governance, equity, and institutional trust [4,26]. Our observation that sociopolitical reactions dominated comment threads regardless of original post topic is consistent with this pattern and highlights how audience engagement can reshape discourse beyond initial informational intent.

The relationship between sentiment and engagement observed here also parallels findings from studies of COVID-19 and other health crises, where negative or controversial content was more likely to attract interaction [33]. Such dynamics may amplify emotionally salient narratives and contribute to the persistence of stigma or misinformation in online environments [34]. However, our analysis further demonstrates that technically oriented posts, particularly those focused on scientific or policy debates, were associated with relatively more positive audience responses, suggesting that constructive discourse can emerge when information is framed in evidence-based contexts.

Overall, these results complement recent research by demonstrating not only the thematic structure of Mpox discussions but also the divergence between original post content and audience responses. This interactional perspective provides additional insight into how public discourse evolves during health emergencies and underscores the importance of anticipating audience-driven reframing in public health communication strategies [35].

### Limitations

However, several limitations should be noted when interpreting these findings. First, the data were drawn exclusively from Reddit, which is mostly popular in North America and other English-speaking countries. Therefore, the results may not reflect the global landscape of Mpox discourse or the communication norms of other platforms. Second, only 4 subreddits were included, and r/Monkeypox [11] contributed a disproportionately large number of posts and comments. This imbalance could bias the observed patterns toward the norms of that particular community, potentially underrepresenting the perspectives seen in more generalist spaces. Third, engagement was measured using Reddit scores (upvotes minus downvotes), which capture one form of audience response, but do not fully represent other interaction behaviors such as commenting, sharing, or passive viewing. Fourth, engagement differences by sentiment may be influenced by unmeasured factors such as author visibility, posting frequency, or source type (eg, personal narratives vs news links), which were not explicitly controlled for in the analysis. Although no individual author contributed more than 25 posts out of the 1169 total (2.1% of the dataset), suggesting that no single author dominated the dataset, residual confounding by author-level or source-level characteristics cannot be ruled out. Fifth, the long temporal range of the dataset (2021‐2025) spans both the early emergency phase of the outbreak and later periods of reduced transmission. As a result, the analysis may conflate discourse dynamics that differ between acute crisis communication and more endemic-phase discussion. Sixth, keyword-based filtering was used to identify Mpox-related posts, which may have omitted relevant discussions occurring in broader news or general-interest subreddits that did not explicitly use the selected terms.

In addition, concise topic theme labels were generated with assistance from an LLM after statistical topic assignments were finalized. While the clustering itself was produced using LDA, the summarization step introduces some degree of stochasticity and subjectivity inherent to LLM-generated outputs. To improve stability, the summarization process was repeated multiple times using different random samples of documents per topic, and the resulting summaries were aggregated, though the themes were not independently validated by multiple human coders or alternative models.

Furthermore, due to computational and token limitations, the LLM-based interpretation step relied on randomly sampled subsets of documents from each topic rather than the full set of posts and comments assigned to that topic. Although these samples were drawn repeatedly across multiple runs to capture representative content, a small proportion of topic-related messages may not have been included in the summaries used to derive the final theme labels.

Finally, sentiment analysis relied on a lexicon-based approach, which may not fully capture sarcasm, irony, or context-specific language commonly found in Reddit discussions. Such nuances may affect the precision of sentiment classification.

While these limitations restrict the generalizability of the findings, they also highlight the value of Reddit as a lens into globally circulating narratives, concerns, and framings of Mpox that may cross national boundaries.

### Conclusion

This study investigated how audience engagement relates to the sentiment and topic types of Reddit posts about Mpox (RQ 1) and how the sentiment and topical focus of comments vary in response to posts of different sentiments and topics (RQ 2). The analysis revealed that engagement was unevenly distributed, with negative posts and those focused on systemic public health failures or sociopolitical reactions receiving higher scores, while posts emphasizing technical details or emerging variants drew lower engagement. Although neutral comments were most common, positive posts tended to elicit more positive comments and negative posts more negative comments. In addition, the focus of comments often differed from that of the original post, with sociopolitical discussions dominating regardless of the post topic.

These findings highlight the complex ways in which public discourse on health crises evolves from initial posts to audience responses. Emotionally charged and blame-oriented content appears more likely to attract engagement, while subsequent discussions frequently reframe posts around sociopolitical concerns. Understanding these dynamics can inform public health communication strategies by helping practitioners design messages that anticipate likely audience reactions, mitigate panic, stigma, and misinformation, as well as foster constructive dialogue during outbreaks. Future research could build on these findings by examining other platforms and expanding the scope beyond Reddit to capture more diverse public perspectives.

## Abbreviations

JSD: Jensen-Shannon divergence
LDA: latent Dirichlet allocation
LGBTQ+: lesbian, gay, bisexual, transgender, queer, and more
LLM: large language model
RQ: research question
